# Plant height is the main factor driving forage yield of *Poa* species under different row spacings and seeding rates in the Qilian Mountains

**DOI:** 10.3389/fpls.2025.1535937

**Published:** 2025-02-21

**Authors:** Xiaojun Wang, Jinlan Wang, ERenCuo Li, Yani Guo, Wen Li

**Affiliations:** ^1^ Qinghai Provincial Key Laboratory of Adaptive Management on Alpine Grassland, Qinghai University, Xining, China; ^2^ Key Laboratory of Development of Forage Germplasm in the Qinghai-Tibetan Plateau of Qinghai Province, Academy of Animal Science and Veterinary, Qinghai University, Xining, China; ^3^ State Key Laboratory of Plateau Ecology and Agriculture, Qinghai University, Xining, China; ^4^ Qinghai Province Sanjiang Technology Company Limited, Xining, China; ^5^ Gansu Materias Group Company Lanzhou Logistics Park Co., Ltd., Lanzhou, China

**Keywords:** cultivation measures, field management, agronomic traits, yield effect, comprehensive evaluation

## Abstract

Scientific and reasonable planting densities are crucial for *Poa* species forage production. However, the optimal row spacing and seeding rate for *Poa* species cultivation, and the effects of row spacing and seeding rate on *Poa* species forage yield in the Qilian Mountains remain unclear. In the current study, *P. sinoglauca* Ohwi., *P. crymophila* Keng, *P. pratensis* L. var. *anceps* Gaud, and *P. pagophila* Bor were selected as study materials, and a split field experiment designed with row spacing as the main factor and seeding rates as the sub-factor was conducted to study the effects of different row spacings and seeding rates on the agronomic traits and forage yield benefits of *Poa* species. The main plots were designed with different row spacings of 15 cm (R_1_), 30 cm (R_2_), and 45 cm (R_3_), and the subplots were designed with different seeding rates of 7.0 kg·hm^-2^ (S_1_), 12.0 kg·hm^-2^ (S_2_), and 17.0 kg·hm^-2^ (S_3_). A subsection structural equation model was used to analyze the influence process and path coefficients of row spacing, seeding rate, and the interaction between row spacing and seeding rate on the yield of *Poa* species, and the Technique for Order Preference by Similarity to an Ideal Solution (TOPSIS) was used to comprehensively assess the agronomic traits and forage yield of the experimental varieties. Our results showed that row spacing significantly affected the plant height, tiller number, fertile tiller number, and forage yield of the four *Poa* species, while seeding rate and interaction between row spacing and seeding rate had significant effects on the forage yield of the four *Poa* species. The highest forage yields of *P. sinoglauca* (6709.1 kg·hm^-^²) and *P. crymophila* (7471.3 kg·hm^-^²) were recorded for a row spacing of 30 cm and seeding rate of 17 kg·hm^-^², and the highest forage yields of *P. pratensis* L. var. *anceps* (9469.0 kg·hm^-^²) and *P. pagophila* (8152.7 kg·hm^-^²) were recorded for a row spacing of 30 cm and seeding rate of 12 kg·hm^-^². Structural equation modeling indicated that row spacing, seeding rate, and the interaction between row spacing and seeding rate primarily affected the forage yield of *Poa* species by affecting plant height. Our research provided optimal sowing and row spacing for *Poa* species depending on the species to optimize forage production in the southern Qilian Mountains and similar areas.

## 1 Introduction

The Qilian Mountains are characterized by diverse terrain, crisscrossing rivers, and abundant grassland resources and represent one of China’s vital livestock production bases (Du et al., 2024). Moreover, they serve as a natural barrier that safeguards the ecological security in western China (Baranova et al., 2016). However, in recent years, owing to global warming and human disturbance, grasslands in the Qilian Mountains have been severely degraded, resulting in decreased forage production and significant forage-livestock conflicts, which have hindered the sustainable development of livestock husbandry in this region (Deng, 2022). Therefore, enhancing degraded grassland restoration and management, increasing forage production, and resolving forage-livestock conflicts are urgent tasks. Establishing perennial artificial grasslands improves grassland productivity, enhances forage quality, alleviates forage-livestock conflicts, improves the soil structure, increases soil moisture content and fertility, and provides strong ecological services (Sanford et al., 2022). This approach represents an effective method of restoring degraded grasslands (Song et al., 2023). *Poa* species belongs to the Gramineae family and is characterized by high adaptability, abundant leaf mass, tender forage, rich nutrition, good palatability, strong soil conservation and water retention capabilities (Zhou et al., 2008); thus, it represents an excellent forage species for establishing artificial grasslands. As a dominant grass species in the Qilian Mountain region, *Poa* species play a crucial role in ecological management, soil and water conservation, and grassland livestock husbandry development (Zhou et al., 2014). However, owing to the climatic conditions of the Qilian Mountains and the lack of cultivation techniques for *Poa* species, the forage production of *Poa* species in this region is low, and economic benefits are limited, significantly impacting the sustainable development of grassland livestock husbandry.

Scientific cultivation techniques effectively realize forage production potential (Zhu et al., 2024). Forage production is mainly affected by the planting row spacing (Baath et al., 2024), seeding rate (Beck et al., 2022), sowing date (Jung et al., 2023), seeding ratio (Wang et al., 2024a), and fertilization rate (Mattera et al., 2023). Hua et al. (2023) showed that hay yield and seed yield of *Poa* pratensis L. Qinghai was obtained at a row spacing of 20 cm and sowing amount of 5 kg·hm^-2^ in Guinan county of Qinghai province. Wang et al. (2022) indicated that the highest seed yield of *Bromus inermis* was recorded for a row spacing of 30 cm and seeding rate of 15 kg·hm^-^². Jing et al. (2023) showed that the optimal aboveground biomass and root surface area of *P. annua* L. ‘Qinghai’ were reached at a seeding density of 6478 seeds·m^-^², whereas the optimal number of underground buds was observed at a row spacing of 15 cm and a seeding density of 2782 seeds·m^-^². Wang et al. (2024a) reported that a reasonable increase in planting density significantly enhanced potassium accumulation in the plant community by facilitating potassium absorption, accumulation, and translocation in the canopy leaves and stems. When the planting density of forage exceeds the optimal value, environmental factors such as soil fertility and light become insufficient to support the growth and development of all plants. This intensifies intraspecific competition, weakens stem growth, and increases forage lodging susceptibility but also delays the flowering period, hindering insect pollination and affecting seed yield (Wang et al., 2023; Wei, 2023). Conversely, when the planting density of forage is lower than the optimal density, forage cannot fully utilize environmental resources, leading to decreased forage production (Aklilu, 2020). Thus, the planting density and environmental resources must be effectively balanced to maximize the productivity of grasslands.

Scientific and reasonable planting densities are crucial for *Poa* species forage production. However, the optimal row spacing and seeding rate for *Poa* species cultivation in the Qilian Mountains remain unclear, and the effects of row spacing and seeding rate on *Poa* species forage yield have not been clarified. This gap constrains the sustainable development of grassland livestock husbandry in the region, highlighting the urgent need for research on the most suitable row spacing and seeding rate for *Poa* species cultivation. *P. sinoglauca* Ohwi. and *P. crymophila* Keng are rhizomatous grasses (Li et al., 2001; Liu, 2009.), while *P. pratensis* L. var. *anceps* Gaud and *P. pagophila* Bor are perennial rhizome type (Shi et al., 2017; Yao et al., 2009). All four *Poa* species have a wide range of adaptability, excellent resistance to adverse conditions, high nutritional value, easy management, and ecological friendliness, making them suitable for planting in the Tibetan Plateau (Li et al., 2022). However, *P. sinoglauca* and *P. pratensis* var. *anceps* have slow seedling growth and are easily suppressed by weeds (Huang and Zhang, 2021); the growth and development of *Poa crymophila* and *Poa pagophila* are strongly affected by biological and abiotic factors (Ma et al., 2009). In view of the significant differences in the feedback of the four *Poa* species to environmental factors, *P. sinoglauca*, *P. crymophila*, *P. pratensis* var. *anceps*, and *P. pagophila* were selected as test materials in this study to study the effects of different planting row spacing and seeding rates on the forage yield and agronomic traits. The objective of this study was to evaluate the agronomic traits and forage yield to determine how *P. annua* responds to different row spacing and seeding rate combinations and provide data support for *Poa* species forage production in this region and similar areas.

## 2 Materials and methods

### 2.1 Study area

**Figure 1:**
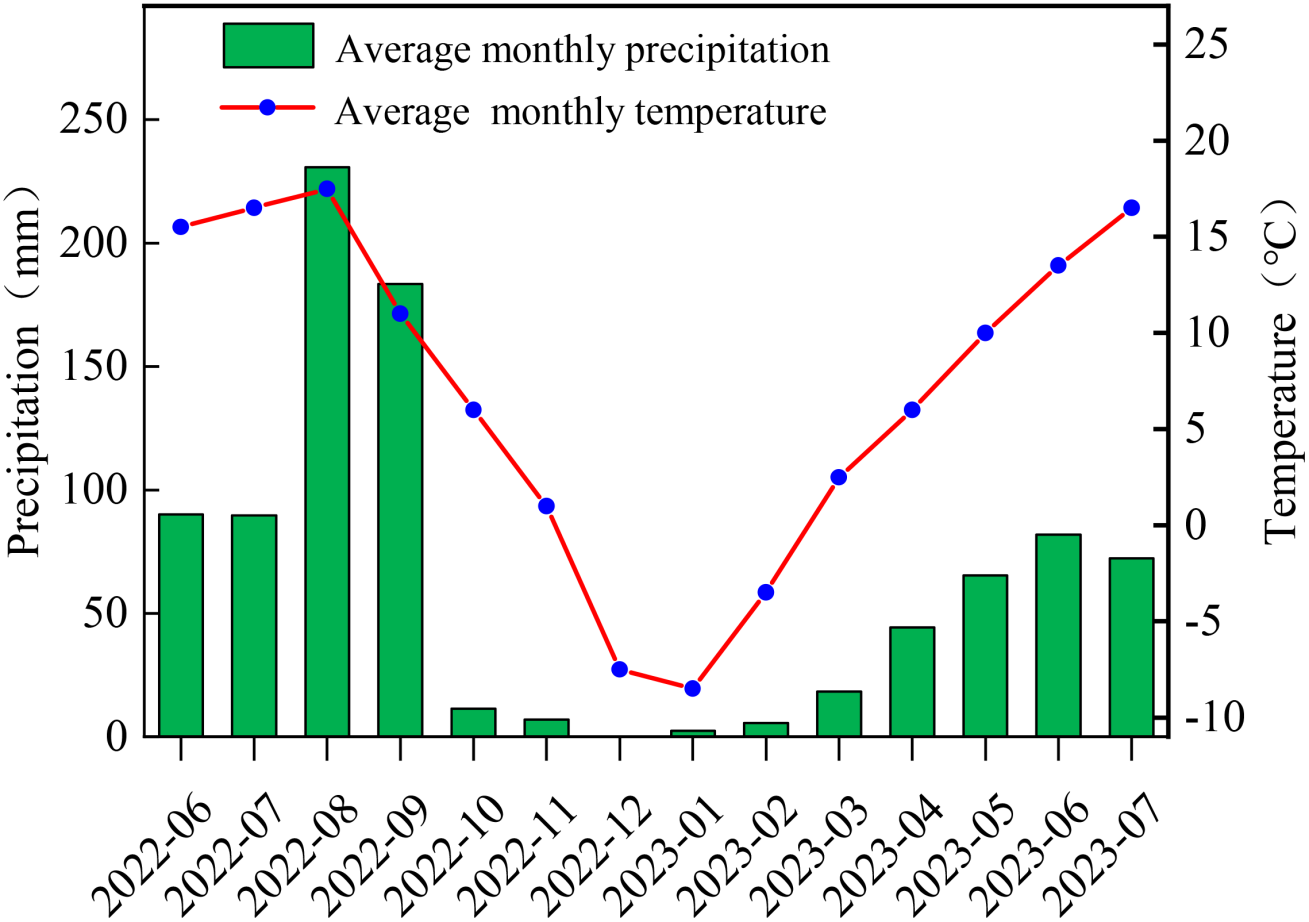
Distribution of average monthly precipitation and temperature from June 2022 to July 2023 at the study site.

The experimental site (36°59′ N, 100°18′ E) is located in Datong Hui Autonomous County, Xining City, Qinghai Province, at an altitude of 3180 m. The area is characterized by a typical plateau continental climate, with abundant sunshine, strong solar radiation, and significant diurnal temperature variations. The annual frost-free period ranges from approximately 61 to 133 d, and the average annual sunshine duration is 2553 h. The annual mean temperature is 4.9°C, with an average annual precipitation of 523.3 mm and an annual evaporation of 1762.8 mm. Precipitation is highest in August and lowest in December, with an average annual relative humidity of 56%. From June 2022 to July 2023, the highest and lowest monthly mean temperatures at the experimental site were 17.5°C and -8.5°C, respectively, while the highest and lowest monthly precipitation values were 230.7 mm and 0 mm, respectively (**Figure 1**). The soil type at the experimental site is a chernozem, the soil organic matter is 65.12 g·kg^-1^, total nitrogen is 3.47 g·kg^-1^, total phosphorus is 1.21 g·kg^-1^, total potassium is 20.11 g·kg^-1^, available nitrogen is 60.34 mg·kg^-1^, available phosphorus is 5.75 mg·kg^-1^, and available potassium is 120.17 mg·kg^-1^.

### 2.2 Experimental materials

The test materials consisted of the four *Poa* species: *P. sinoglauca*., *P. crymophila*, *P. pratensis* L. var. *anceps*, and *P. pagophila*, which were provided by the Qinghai Academy of Animal Science and Veterinary Medicine. The basic characteristics of each species are listed in **Table 1**.

**Table 1:** The basic characteristics of tested *Poa* species.

Number	Species	Seed purity (%)	Germination Rate (%)	true value of seeds (%)
1	*P. sinoglauca* Ohwi.	93.3	86.6	80.8
2	*P. crymophila* Keng	93.5	85.2	79.7
3	*P. pratensis* L. var. *anceps* Gaud	90.8	88.5	80.4
4	*P. pagophila* Bor	91.9	84.7	77.8

### 2.3 Experimental design

A split-plot experimental design was used. Three-row spacing treatments and three seeding rate treatments were set up as the main factor and sub-factor: 15 cm (R_1_), 30 cm (R_2_), and 45 cm (R_3_); 7.0 kg·hm^-^² (S_1_), 12.0 kg·hm^-^² (S_2_), and 17.0 kg·hm^-^² (S_3_), respectively. These treatments were based on the Qinghai provincial standards DB 63/T 934-2010 and DB 63/T 1915-2021. Moreover, the row spacing and seeding rates for planting *P. pratensis* L. var. *anceps* was based on the research of Jing et al. (2023) and taking account the seed value of 100% according to the data in **Table 1** when determining the seeding rate. Thus, there were nine treatments and three repetitions, with 27 plots for each species and 108 plots for the four species. In addition, the area was 15 m^2^ (3 ^m^ ×5 m), spacing between areas was 1 m, and spacing between blocks was 3 m. Sowing was performed in June 2022 using the drill seeding method with the sowing depth 2 cm. Before sowing, urea (46% N content) and superphosphate (20% P_2_O_5_ content) were applied as base fertilizers, with each providing 60 kg·hm^-^² of nitrogen (N) and phosphorus (P_2_O_5_), respectively. After sowing, no irrigation or covering measures were applied, and manual removal of impurities was selected, and no irrigation, fertilization, and other measures were taken since then. After 30 days of sowing, the number of seedlings in the field was investigated, and then divided by the number of seeds × 100 to calculate the field emergence rates. And the field emergence rates of *P. sinoglauca*, *P. crymophila*, *P. pratensis* L. var. *anceps* and *P. pagophila* were 80.3%, 82.5%, 84.7% and 82.1%, respectively.

### 2.4 Field survey and sampling

In late July 2023, indicator measurements and sample collection commenced when 80% of the *Poa* species plants reached flowering time after the border rows and 50 cm at each end of the experimental plots were manually removed to eliminate edge effects. First, ten plants with consistent growth status and no obvious pests or diseases were randomly selected from each plot to measure the absolute height using steel tape, and the stem diameter was measured at the second stem node using a Vernier caliper. Six 1-meter segments with relatively consistent growth were randomly selected from each experimental plot, and the number of tillers and fertile tillers was measured after cutting at ground level, followed by separation of the stems and leaves. Finally, three 1 m × 1 m quadrats were randomly selected in each plot. All plants in the quadrat were cut at ground level and weighed immediately after removing weeks, and the fresh grass yield was recorded. The samples were collected in envelopes and brought back to the laboratory, dried at 105°C for 30 min, and then transferred to a 75°C oven and dried to a constant weight. The weight was recorded and used to calculate the stem-to-leaf ratio (stem dry weight/leaf dry weight), dry-to-fresh weight ratio, and dry herbage yield.

### 2.5 Statistical analysis

Data were initially organized using Microsoft Excel 2021, and the normality and homogeneity of variances were tested using SPSS 19.0 (Chicago, IL, USA) software. One-way ANOVA of the agronomic traits and forage yield under different row spacings and seeding rates was performed using SPSS 19.0, and two-way ANOVA of the effect of the interaction between row spacing and seeding rate on the agronomic traits and forage yield of each *Poa* species was performed at the 0.05 significance level.

To identify the optimal row spacing and seeding rate for forage production of *Poa* species in the Qilian Mountains and similar regions, TOPSIS was used to comprehensively evaluate the agronomic traits and forage yield of each *Poa* species using the plyr package in R 4.3.1 (R Development Core Team). Mantel test analysis (dplyr R package in R 4.3.1) was used to explore the correlations between agronomic traits and forage yield for the four *Poa* species, and the PiecewiseSEM package was used to construct piecewise structural equation models to investigate the processes and path coefficients of the effects of row spacing, seeding rate, and the interactions between row spacing and seeding rate on the forage yield of *Poa* species. Graphs were created using Origin 2019 software, and the results are expressed as the “mean ± standard error” in the figures and tables.

## 3 Results

### 3.1 Effects of different row spacings and seeding rates on the agronomic traits of *Poa* species

**Figure 2:**
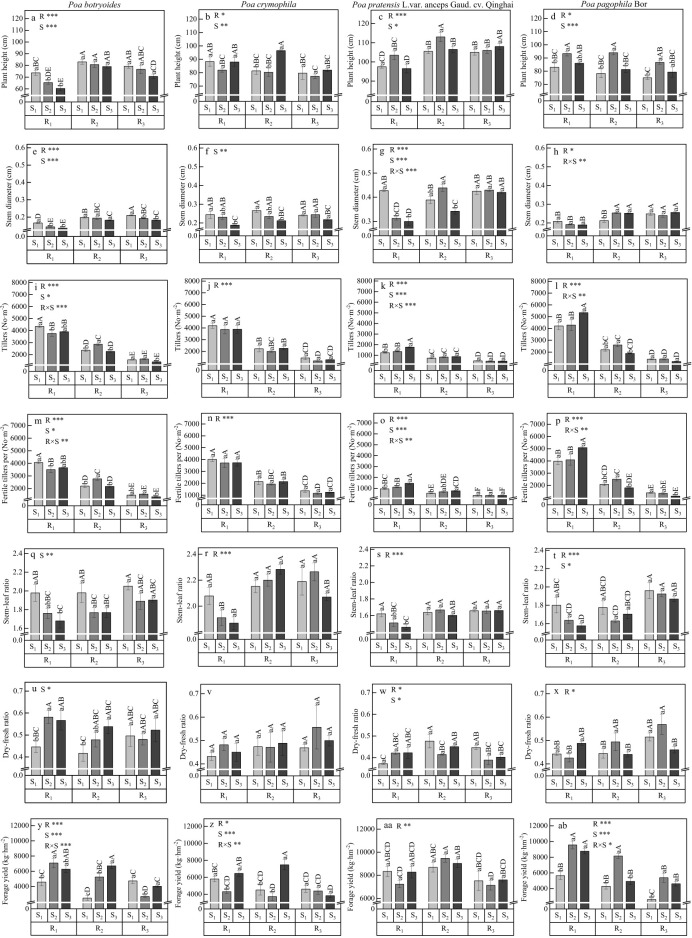
Effects of different row spacings and seeding rates on the agronomic traits of different *Poa* species. R represents row spacing, S represents seeding rate, and R × S represents the interaction between row spacing and seeding rate. *P<0.05, **P<0.01, ***P<0.001. Different lowercase letters indicate significant differences among sowing rates at the same row spacing (*P*<0.05); and different capital letters indicate significant differences among all treatments (*P*<0.05); the same below. Figures **(a, e, i, m, q, u, y)** represent the plant height, stem diameter, tiller count, number of fertile tillers per plant, stem-to-leaf ratio, dry-to-fresh weight ratio, and forage yield of *Poa sinoglauca*; Figures **(b, f, j, n, r, v, z)** represent the plant height, stem diameter, tiller count, number of fertile tillers per plant, stem-to-leaf ratio, dry-to-fresh weight ratio, and forage yield of *Poa crymophila*; Figures **(c, g, k, o, s, w, aa)** represent the plant height, stem diameter, tiller count, number of fertile tillers per plant, stem-to-leaf ratio, dry-to-fresh weight ratio, and forage yield of *Poa pratensis* L.var. *anceps*; Figures **(d, h, l, p, t, x, ab)** represent the plant height, stem diameter, tiller count, number of fertile tillers per plant, stem-to-leaf ratio, dry-to-fresh weight ratio, and forage yield of *Poa pagophila*.

As shown in **Figure 2**, row spacing had a mostly significant effect on the plant height, stem diameter, tiller, fertile tiller number, stem-leaf ratio, dry-fresh ratio and forage yield (except for stem-leaf ratio and dry-fresh ratio of *P. sinoglauca*, and stem diameter and dry-fresh ratio of *P. crymophila*) among the four species. The seeding rate significantly affected the plant height among the four species, a significant effect on the stem diameters of *P. sinoglauca*, *P. crymophila*, and *P. pratensis* L. var. *anceps*, a significant effect on the tillers, the fertile tiller number and dry-fresh radio of the *P. sinoglauca* and *P. pratensis* L. var. *anceps*, a significant effect on the stem-leaf ratio of the *P. sinoglauca* and *P. pagophila*, and a significant effect on the forage yield of the four *Poa* species. The interaction between row spacing and seeding rate significantly affected on the stem diameters of the *P. pratensis* L. var. *anceps* and *P. pagophila*, a significant effect on the tiller and fertile tiller number of *P. sinoglauca*, *P. pratensis* L. var. *anceps*, and *P. pagophila*, and had a significant effect on the forage yield of the four *Poa* species (except for *P. pratensis* L. var. *anceps*).

For *P. sinoglauca*, the plant height was the highest in the R_2_S_1_, R_2_S_2_, R_2_S_3_, R_3_S_1_ and R_3_S_2_ treatments at 76.7–83.0 cm (**Figure 2a**), the stem diameters was highest in the R_3_S_1_ treatment at 0.21 cm (**Figure 2e**), the tillers and fertile tillers per was highest in the R_1_S_1_ treatment at 4354.4 No·m^-2^ and 4062.2 No·m^-2^ (**Figures 2i, m**), the stem-leaf ratio was highest in the treatments R_1_S_1_, R_2_S_1_, R_3_S_1_, R_3_S_2_, R_3_S_3_ at 1.89–2.05 (**Figure 2q**), the dry-fresh ratio was highest in the R_1_S_2_, R_1_S_3_, R_2_S_2_, R_2_S_3_, R_3_S_1_, R_3_S_2_ and R_3_S_3_ treatments at 0.48–0.58 (**Figure 2u**), and the forage yield was highest in the R_1_S_2_, R_1_S_3_ and R_2_S_3_ treatments at 6271.1–7079.5 kg·hm^-2^ (**Figure 2y**). For *P. crymophila*, the highest plant was height in the R_1_S_1_, R_1_S_3_ and R_2_S_3_ treatments at 88.0–96.5 cm, which was significantly higher than that R_1_S_2_, R_2_S_1_, R_2_S_2_, R_3_S_1_, R_3_S_2_ and R_3_S_3_ (**Figure 2b**), the highest stem diametes were observed in the R_1_S_1_, R_1_S_2_, R_2_S_1_, R_2_S_2_, R_3_S_1_ and R_3_S_2_ treatments at 0.23–0.27 cm, which was significantly higher than that in R_1_S_3_, R_2_S_3_ and R_3_S_3_ (**Figure 2f**), the highest tillers and fertile tillers per were observed in the R_1_S_1_, R_1_S_2_, R_1_S_3_ and R_3_S_2_ treatments at 3871.1–4196.7 No.m^-2^ and 3715.6–4024.4 No.m^-2^, which was significantly higher than that in R_2_S_1_, R_2_S_2_, R_2_S_3_, R_3_S_1_, R_3_S_2_ and R_3_S_3_ (**Figures 2j, n**), the highest stem-leaf ratios were observed in the R_1_S_1_, R_2_S_1_, R_2_S_2_, R_2_S_3_, R_3_S_1_, R_3_S_2_ and R_3_S_3_ treatments at 2.08–2.28, which was significantly higher than that in R_1_S_2_ and R_1_S_3_ treatments (**Figure 2r**), but row spacing and seeding rate have no significant effect on the dry-to-fresh ratio (**Figure 2v**). In addition, the highest forage yield was observed in the R_1_S_3_ and R_2_S_3_ treatments at 6465.6–7471.3 kg·hm^-2^, which was significantly higher than that in the R_1_S_1_, R_1_S_2_, R_2_S_1_, R_2_S_2_, R_3_S_1_, R_3_S_2_, and R_3_S_3_ treatments (**Figure 2z**). For *P. pratensis* L. var. *anceps*, the highest plant height was observed in the R_2_S_2_ and R_3_S_3_ treatments at 108.0–113.0 cm, which was significantly higher than that of the other treatments (**Figure 2c**), the highest stem diameters was the observed in the R_1_S_1_, R_2_S_2_, R_3_S_1_, R_3_S_2_, and R_3_S_3_ treatment at 0.42–0.44 cm, which was significantly higher than that in the R_1_S_2_, R_1_S_3_, R_2_S_1_, and R_2_S_3_ treatments (**Figure 2g**), the tillers and fertile tillers per was highest in the R_1_S_3_ treatments at 1770.0 No.m^-2^ and 1458.3 No.m^-2^ (**Figures 2k, o**). In addition, the higher stem-leaf ratios were observed in the R_1_S_1_, R_2_S_1_, R_2_S_2_, R_2_S_3_, R_3_S_1_, R_3_S_2_ and R_3_S_3_ treatments at 1.60–1.67 (**Figure 2s**) and the highest dry-to-fresh ratio were observed in the R_1_S_2_, R_1_S_3_, R_2_S_1_, R_2_S_3_ and R_3_S_1_ treatments at 0.41–0.48, which was significantly higher than that in the R_1_S_1_, R_2_S_2_, R_3_S_2_ and R_3_S_3_ treatments (**Figure 2w**); while the highest forage yield was observed in the R_1_S_1_, R_1_S_3_, R_2_S_1_, R_2_S_2_ and R_2_S_3_, treatment at 8276.0–9469.0 kg·hm^-2^, which was significantly higher than that in the R_1_S_2_, R_3_S_1_, R_3_S_2_, and R_3_S_3_ treatments (**Figure 2aa**). For *P. pagophila*, the plant height was highest in the R_1_S_2_, R_1_S_3_, R_2_S_2_ and R_3_S_2_ treatments at 86.0–94.0 cm, which was significantly higher than that in the R_1_S_1_, R_2_S_1_, R_2_S_3_, R_3_S_1_, and R_3_S_3_ treatments (**Figure 2d**), the highest stem diameters was observed in the R_2_S_2_, R_2_S_3_, R_3_S_1_, R_3_S_2_ and R_3_S_3_ treatments at 0.24–0.26 cm, which was significantly higher than that in the R_1_S_1_, R_1_S_2_, R_1_S_3_, and R_2_S_1_ treatments (**Figure 2h**), the highest tillers and fertile tillers per was observed in the R_2_S_1_ treatments at 5337.8 No.m^-2^ and 5090.0 No.m^-2^, which was significantly higher than that of the other treatments (**Figures 2l, p**). In addition, the highest stem-leaf ratios were observed in the R_1_S_1_, R_2_S_1_, R_3_S_1_, R_3_S_2_ and R_3_S_3_ treatments at 1.78–1.96, which was significantly higher than that in the R_1_S_2_, R_1_S_3_, R_2_S_2_ and R_2_S_3_ treatments (**Figure 2t**) and the highest dry-fresh ratios were observed in the R_1_S_3_, R_2_S_2_, R_3_S_1_ and R_3_S_2_ treatments at 0.49–0.57, which was significantly higher than that in the R_1_S_1_, R_1_S_2_, R_2_S_1_, R_2_S_3_ and R_3_S_3_ treatments (**Figure 2x**); while the highest forage yield was observed in the R_1_S_2_, R_1_S_3_, and R_2_S_2_ treatments at 8152.7–9558.1 kg·hm^-2^, which was significantly higher than that in the R_1_S_1_, R_2_S_1_, R_2_S_3_, R_3_S_1_, R_3_S_2_, and R_3_S_3_ treatments (**Figure 2ab**).

### 3.2 Comprehensive evaluation of four *Poa* species under different row spacings and seeding rates

**Figure 3:**
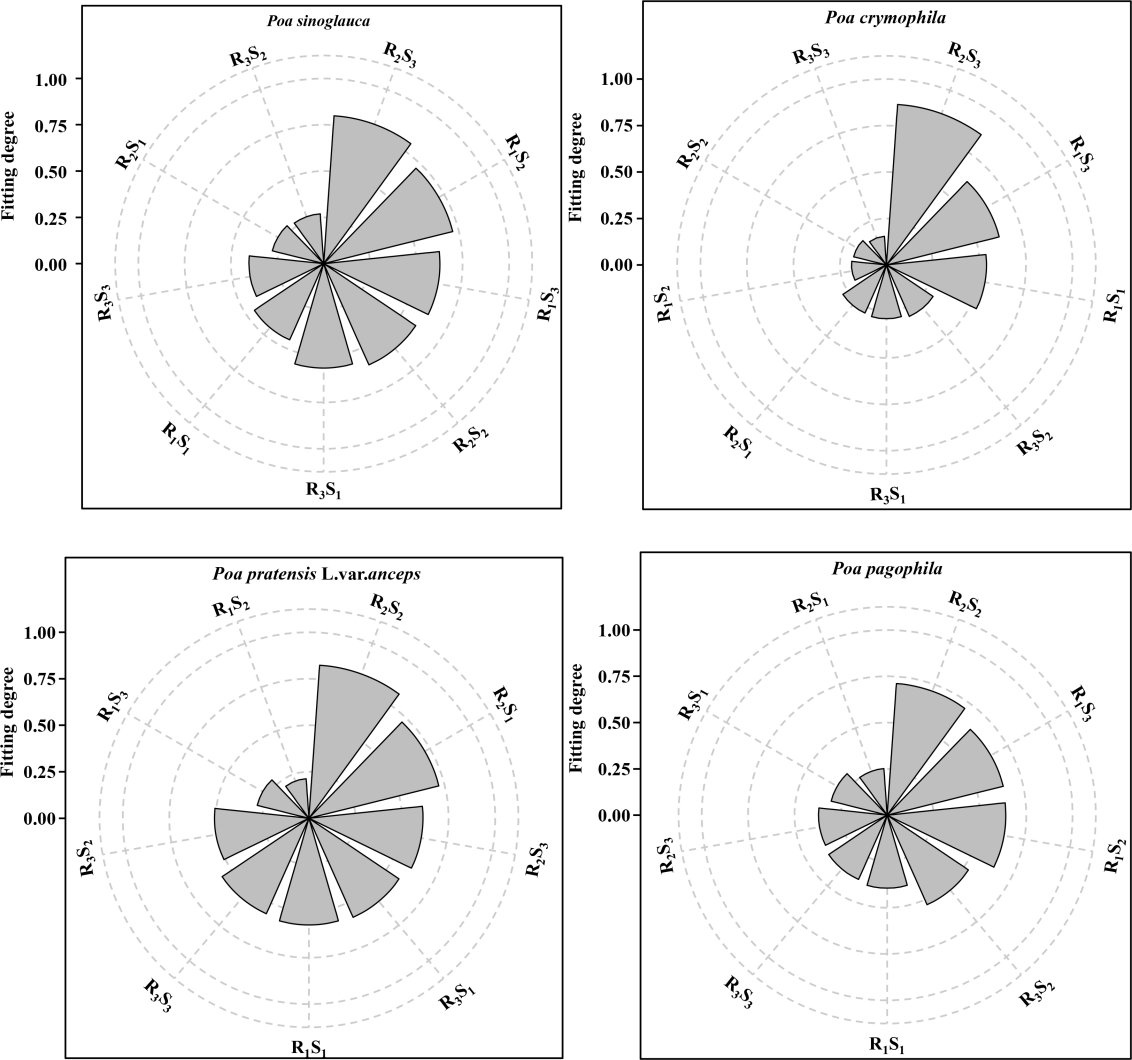
Comprehensive evaluation of forage yield of *Poa* species under different row spacings and seeding rates.

TOPSIS was used to comprehensively evaluate the plant height, stem diameter, stem-leaf ratio, dry-fresh ratio, and forage yield of the four *Poa* species under different row spacings and seeding rates (**Figure 3**). The results indicated that *P. sinoglauca* and *P. crymophila* had the highest fitness in the R_2_S_3_ treatment, with values of 0.80 and 0.87, respectively; while *P. pratensis* L. var. *anceps* and *P. pagophila* had the highest fitness in the R_2_S_2_ treatment, with values of 0.83 and 0.71, respectively. Therefore, the forage yield of *Poa* species was significantly improved in the R_2_S_3_ treatment for *P. sinoglauca* and *P. crymophila* and the R_2_S_2_ treatment for *P. pratensis* L. var. *anceps* and *P. pagophila*, making them the optimal row spacings and seeding rates for forage production of *P. annua* in the Qilian Mountains.

### 3.3 Correlation analysis between the forage yield and agronomic traits of the four *Poa* species

**Figure 4:**
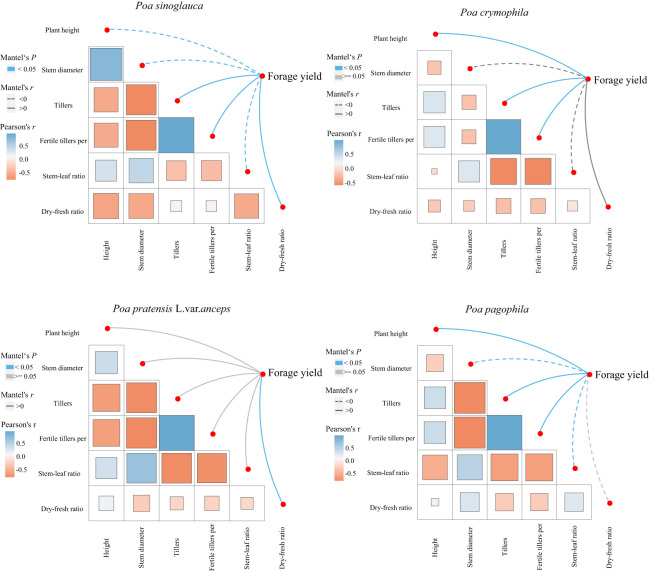
Mantel test analysis between the yield and performance of the four *Poa* species.

Mantel test analysis indicated that the forage yield of *P. sinoglauca* was significantly positively correlated with the tiller and fertile tiller number and dry-fresh ratio and significantly negatively correlated with the plant height, stem diameter, and stem-leaf ratio (**Figure 4**). The forage yield of *P. crymophila* was significantly positively correlated with plant height and tiller and fertile tiller number; that of *P. pratensis* L. var. *anceps* was significantly positively correlated with the dry-fresh weight ratio; and that of *P. pagophila* was significantly positively correlated with the plant height and tiller and fertile tiller number and significantly negatively correlated with the stem diameter and stem-leaf ratio.

### 3.4 Mechanisms underlying the effects of row spacing and seeding rate on the forage yield of *Poa* species

**Figure 5:**
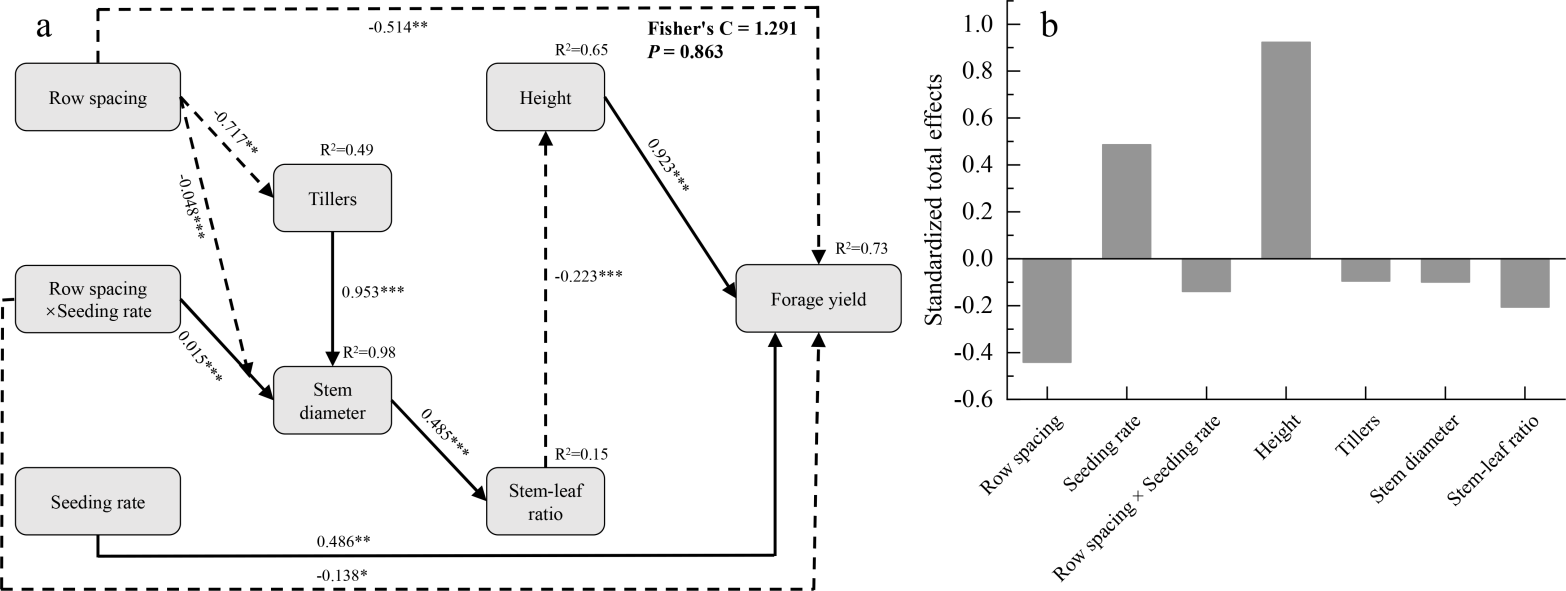
Structural equation model analysis of the effects of row spacing and seeding rate on the forage yield of *Poa* species **(A)**. Total effects of row spacing and seeding rate on forage yield of *Poa* species **(B)**. Black solid and dashed arrows represent significantly positive or negative effects at the 0.05 level, respectively. *P<0.05, **P<0.01, ***P<0.001.

A piecewise structural equation model was used to explore the mechanisms underlying the effects of row spacing, seeding rate, and their interaction on the forage yield of *Poa* species based on agronomic traits (**Figure 5A**). The results showed that the model had a good fit, with a *P*-value of 0.863 and a Fisher’s C value of 1.291. Row spacing, seeding rate, interaction between row spacing and seeding rate, and plant height directly affected the forage yield of *Poa* species, with path coefficients of -0.514, 0.486, -0.138, and 0.923, respectively. Row spacing, and the interaction between the rowing spacing and seeding rate also indirectly affected the forage yield of *Poa* species by influencing the tiller number, stem diameter and stem-leaf ratio. Based on the standard path coefficients in the segmented structural equation, we calculated the total effects on the forage yield of *Poa* species and observed that plant height had the highest total effect value of 0.923 (**Figure 5B**). Therefore, row spacing, seeding rate, and their interaction primarily influenced the forage yield of *Poa* species by impacting plant height.

## 4 Discussion

Scientific and reasonable cultivation measures can greatly exploit the genetic advantages of forage grasses, thereby improving their yield and quality (Rye et al., 2023). In the present study, all four *Poa* species were sampled in the flowering stage at the end of July to monitor the forage yield, which is inconsistent with previous research who addressed that pre-flowering harvesting is generally recommended for grasses with the highest yield of fresh grass (Yue et al., 2024). This maybe own to the special geographical and topographic features of experimental research. *Poa* species were all in the flowering stage in our research with the preparation for seed growth and development and reproductive growth begins to transition to vegetative growth, and the above ground stems, leaves and total biomass almost reached the maximum and tended to be stable. Therefore, yield was measured when the aboveground biomass reached the maximum in the flowering stage at the end of July. Row spacing and seeding rate are among the primary cultivation measures in crop production (Wang et al., 2024b) and represent key methods for adjusting crop planting density (Endalkachew, 2020). An appropriate planting density can maximize the utilization of environmental resources, such as water, fertilizer, air, and heat, thereby achieving high-quality and high-yield forage production (Qin et al., 2021). Our research showed that row spacing significantly affected plant height, stem diameter, tiller and fertile tiller number, and forage yield across the four *Poa* species. The seeding rate had a highly significant effect on stem diameter (except for *P. pagophila*) and a significant effect on forage yield. Different row spacings and seeding rates alter the planting density of forage, directly regulating its spatial distribution (Rye et al., 2023; Wang et al., 2024b). When the forage density is low, grassland will have sufficient light and heat resources, prompting the forage to initially increase its canopy width to compete for more light and heat (Umesh et al., 2022). Therefore, forage typically increases tillering to occupy more space and resources. However, large amounts of land, light, heat, water, and fertilizer resources are wasted under a small number of forage bushes per unit area and large plant gaps (Aklilu, 2020). Meanwhile, the roots absorb limited nutrients in the early stage of forage growth, which results in the aboveground part not producing enough photosynthates, further limiting plant height (Du et al., 2019), which reduces the forage yield per unit area. The number of branches per unit area increases as the planting density increases, improving the forage leaf area index, promoting full utilization of environmental resources, and further increasing forage yield (Bainard et al., 2020). However, when the number of branches per unit area of forage exceeds the optimal density, the plant gap decreases, which reduces the ventilation and permeability between plants and limits photosynthesis (Anil et al., 2010). In addition, excessive density promotes intraspecific competition due to insufficient environmental resources, and forage grasses would use more nutrients to increase plant height at the cost of reducing tillering and stem thickness and striving for more light and heat resources (Aklilu, 2020). When the forage height reaches a certain level or enters the late stage of reproductive growth, the forage suffers large-area lodging because the stalk weight is greater than the stalk-carrying capacity, resulting in poor forage development and a decline in grassland productivity (Liu et al., 2024). The results of this study indicated that the forage yield of *P. sinoglauca* and *P. crymophila* was maximized at a row spacing of 30 cm and a seeding rate of 17 kg·hm^-^². For *P. pratensis* L. var. *anceps* and *P. pagophila*, the forage yield was maximized at a row spacing of 30 cm and a seeding rate of 12 kg·hm^-^². This is because, under this row spacing and sowing amount, all four *Poa* species achieved the optimal planting density, optimal spatial allocation, good forage growth and development, full utilization of environmental resources, and highest photosynthetic efficiency, which is conducive to the effective accumulation of dry matter, thus improving the forage yield (Liu et al., 2022).

The results of the piecewise structural equation model showed that row spacing, seeding rate, and their interaction primarily affected the forage yield of *Poa* species by impacting the plant height. Plant height was significantly and positively correlated with the forage yields of *P. crymophila* and *P. pagophila*. This may be because, under optimal planting density, forage can fully utilize environmental resources, achieving the highest leaf photosynthetic efficiency, accelerating plant height growth and development, thereby increasing forage yield (Liu et al., 2022). Our research provided differently optimal sowing and row spacing for *Poa* species, can achieve a high forage yield and thus represent the ideal planting configurations for *Poa* species forage production in the Qilian Mountains. This may be mainly own to the classification of grass groups according to tillering due to their growth habits and reproduction methods for the experimental material, *P. pratensis* L. var. *anceps* Gaud and *P. pagophila* are rhizomatous grasses capable of forming vertical branches and horizontal underground rhizomes during the tillering stage (Lei et al., 2011; Yuan et al., 2022), and new branches continuously grow and gradually develop into new plants, increasing the forage density and decreasing the planting density with increasing years of planting, and the *P. sinoglauca* and *P. crymophila* belongs to the rhizomatous grasses, which grows in sparse clumps with more dispersed tillers, thus forming a sparse plant population (Li et al., 2001; Liu, 2009). Hence, the suitable seeding rate for *P. sinoglauca* and *P. crymophila* was lower than *P. pratensis* L. var. *anceps* and *P. pagophila*. However, our study only analyzed the agronomic traits of 2-year-old *Poa* species under different row spacings and seeding rates, which presents certain limitations regarding the growth and development processes of perennial forages. Continuous observational studies should be conducted over multiple years to comprehensively evaluate the effects of row spacing and seeding rate on the agronomic traits and forage yield of *Poa* species.

## 5 Conclusion

Row spacing significantly affected plant height, tiller and fertile tiller number, and forage yield of the four *Poa* species. The seeding rate and interaction between seeding and rowing spacing significantly influenced the forage yield of the four *Poa* species. *P. sinoglauca* and *P. crymophila* achieved the maximum forage yield at a row spacing of 30 cm and seeding rate of 17 kg·hm^-^², while *P. pratensis* L. var. *anceps* and *P. pagophila* achieved the maximum yield at a row spacing of 30 cm and a seeding rate of 12 kg·hm^-^². Structural equation modeling indicated that row spacing, seeding rate, and the interaction between seeding and rowing spacing primarily affected the yield of *Poa* species via plant height. TOPSIS analysis indicated that row spacings and seeding rates of 30 cm and 17 kg·hm^-^² and 30 cm and 12 kg·hm^-^², respectively, are ideal for *Poa* species forage production in the southern Qilian Mountains, and our research could provide data support for alleviating forage-livestock conflicts.

## Data Availability

The raw data supporting the conclusions of this article will be made available by the authors, without undue reservation.
